# Utilizing [^18^F]-FDG PET/CT Imaging for Enhanced Staging and Treatment Decisions in Pediatric Rhabdomyosarcoma

**DOI:** 10.3390/cancers18101629

**Published:** 2026-05-18

**Authors:** Hadeel Halalsheh, Nada Odeh, Arwa Kiswani, Mohammad Alzoubi, Adam Diab, Noor Al-Assaf, Akram Al-Ibraheem, Ahmad Kh. Ibrahimi, Mohammad Boheisi, Iyad Sultan

**Affiliations:** 1 Department of Pediatrics, King Hussein Cancer Center, Amman 11941, Jordan; 2 School of Medicine, The University of Jordan, Amman 11941, Jordan; 3 Nuclear Medicine Department, King Hussein Cancer Center, Amman 11941, Jordan; 4 Department of Radiation Oncology, King Hussein Cancer Center, Amman 11941, Jordan; 5 Nursing Department, King Hussein Cancer Center, Amman 11941, Jordan

**Keywords:** pediatric rhabdomyosarcoma, [^18^F]-FDG PET/CT, staging, radiotherapy planning, prognosis

## Abstract

Background: Accurate staging is vital for optimizing outcomes in pediatric rhabdomyosarcoma (RMS). While [^18^F]-FDG PET/CT is increasingly utilized, its specific impact on clinical management and its prognostic value compared to conventional imaging (CI) require further evaluation. Methods: In this retrospective single-center study, we reviewed 56 pediatric patients with RMS who underwent [^18^F]-FDG PET/CT at our center. Imaging findings were compared with CI (CT/MRI) and correlated with clinical management and survival outcomes. Results: In the total cohort (*n* = 56), PET/CT demonstrated high concordance with CI for nodal assessment, with an apparent sensitivity of 89.5% and specificity of 94.6%. PET/CT identified skeletal metastases in 5 patients (8.9%) and correctly characterized suspicious pulmonary nodules in one case, though it failed to detect a 0.6 cm lung nodule visualized on chest CT. Notably, PET/CT findings directly altered clinical management in 16.1% of patients (*n* = 9), primarily through radiotherapy adjustments, including field expansions (*n* = 4), field reductions (*n* = 3), and the initiation of previously unplanned radiotherapy (*n* = 2). At a median follow-up of 33.3 months, an exploratory analysis showed that patients with an SUVmax ≥3.6 had a lower 3-year EFS (57.6% vs. 71.6%; *p* = 0.51) and OS (60.4% vs. 71.6%; *p* = 0.63); neither comparison reached statistical significance. Conclusion: [^18^F]-FDG PET/CT is a powerful adjunct in pediatric RMS staging, particularly for nodal and skeletal evaluation. Its ability to refine radiotherapy planning in nearly one-sixth of cases underscores its clinical utility. SUVmax is not a validated prognostic or predictive biomarker in pediatric RMS; prospective, adequately powered multicenter studies, ideally incorporating volumetric PET parameters, are needed before any role in risk-stratified therapy can be defined.

## Simple Summary

Rhabdomyosarcoma is the most common soft tissue cancer in children. Accurately determining the stage of the disease is crucial for choosing the most effective treatment. Traditionally, doctors rely on conventional imaging, such as CT and MRI scans. Our study evaluated whether adding an [^18^F]-FDG PET/CT scan improves this staging process. By reviewing the records of 56 children treated at our center, we found that PET/CT is a highly accurate tool, particularly for detecting disease in lymph nodes and bones. Most importantly, the information provided by the PET/CT scan directly altered the clinical treatment plan for 16.1% of the patients. These changes primarily involved adjusting radiation therapy to be more precise—either by targeting hidden cancer spots or by sparing healthy tissue. We also observed that the metabolic intensity of the tumor on the PET scan (measured as SUVmax) may help predict patient outcomes, though larger studies are needed to confirm this. Ultimately, our findings support the routine use of PET/CT to help personalize and optimize treatment for children with rhabdomyosarcoma.

## 1. Introduction

Rhabdomyosarcoma (RMS) accounts for approximately half of all pediatric soft tissue sarcomas, making it the most common subtype in children [1]. Clinically, RMS presents significant therapeutic challenges due to its aggressive biologic behavior and high metastatic potential. Despite the implementation of intensive, multimodal treatment regimens, the prognosis for patients with advanced-stage disease remains poor [2]. This emphasizes the critical need for improved strategies in early detection, accurate staging, and therapeutic planning to improve overall survival outcomes.

According to guidelines established by the major pediatric groups including Children’s Oncology Group (COG) and the European Pediatric Soft Tissue Sarcoma Study Group (EpSSG), conventional imaging (CI) remains the cornerstone of staging for pediatric RMS [3,4]. Given its superior soft tissue contrast, Magnetic resonance imaging (MRI) is the modality of choice for local staging, allowing for a precise delineation of the primary tumor, its relationship to adjacent structures, and potential perineural or bone marrow involvement. Computed tomography (CT) serves as a complementary tool, particularly for detecting pulmonary metastases and subtle cortical bone erosions, or in situations where MRI is contraindicated or unavailable [3]. For systemic staging, bone scintigraphy and, increasingly, whole-body MRI are used to evaluate distant bone and bone marrow involvement [4,5].

In recent years, [^18^F]-fluorodeoxyglucose Positron Emission Tomography/Computed Tomography [^18^F]-FDG PET/CT) has emerged as a valuable adjunct to CI. By integrating metabolic activity with precise location, PET/CT provides a comprehensive, whole-body evaluation for an active tumor in a single session, often eliminating the need for multiple independent studies such as standalone CTs, bone scintigraphy, and bone marrow assessments. Over the past two decades, this modality has become an important tool in pediatric oncology, though its definitive role in RMS continues to evolve [6,7]. Multicenter studies have demonstrated that [^18^F]-FDG PET/CT frequently outperforms CI in detecting nodal, bone, and bone marrow metastases, occasionally prompting disease upstaging and subsequent alterations in clinical management [8,9]. Furthermore, PET/CT yields critical functional data [10,11]; semi-quantitative measures, particularly the maximum standardized uptake value (SUVmax), reflect tumor metabolism and burden, offering potential prognostic insights into tumor aggressiveness [9,12].

Despite widespread clinical adoption, [^18^F]-FDG PET/CT has not been prospectively validated as an independent prognostic or predictive biomarker in pediatric RMS, and no randomized trial has demonstrated that PET/CT-guided staging or risk stratification translates into improved survival. Any evaluation of PET-derived metabolic parameters, including SUVmax, should therefore be regarded as hypothesis-generating rather than confirmatory.

In light of these considerations, there is a clear need for comparative data to establish the clinical utility of [^18^F]-FDG PET/CT in pediatric RMS. Therefore, this study aims to evaluate the clinical utility of [^18^F]-FDG PET/CT compared to conventional imaging (CI) in the initial staging of pediatric rhabdomyosarcoma (RMS), assess its direct impact on clinical management and radiotherapy planning, and explore the prognostic significance of baseline metabolic parameters (SUVmax) on survival outcomes.

## 2. Patients and Methods

This retrospective cohort study was conducted at King Hussein Cancer Center (KHCC) in Jordan, following approval by KHCC Institutional Review Board (IRB 24 KHCC 98). The study included all pediatric patients (≤18 years) with histologically confirmed RMS who underwent whole-body [^18^F]-FDG PET/CT at initial presentation between May 2016 and December 2023. Patients who received treatment outside (apart from upfront surgery) KHCC were excluded from the analysis. 

Electronic medical records were reviewed to obtain demographics, treatment details, and survival outcomes. Imaging evaluation included both CI and PET/CT. CI, which represents the standard imaging approach for RMS staging, consisted of CT or MRI to assess the primary tumor and chest CT to detect distant metastases. 

Molecular characterization for FOXO1 fusion status was performed via Fluorescence In Situ Hybridization (FISH). While not available for the entire cohort, it was integrated into the routine diagnostic workup from 2019 onward, initially for alveolar subtypes and eventually for all patients.

[^18^F]-FDG PET/CT was acquired according to the current EANM pediatric guidelines [5]. Patients fasted for at least 4–6 h; capillary blood glucose was verified prior to tracer injection; [^18^F]-FDG was administered at a weight-based dose per the EANM pediatric dosage card; uptake time was 60 ± 10 min; and images were acquired from vertex to mid-thigh (or to the feet for lower-extremity primaries). Low-dose CT was used for attenuation correction and anatomic co-registration; images were reconstructed with institutional EARL-compliant parameters. SUVmax was derived from a volume-of-interest placed over the most metabolically active tumor focus. CI was performed per institutional protocols aligned with the COG Diagnostic Imaging Committee/SPR Oncology Committee recommendations [3]. Primary-site imaging used contrast-enhanced MRI, or contrast-enhanced CT; pulmonary evaluation used thin-slice (≤1.5 mm) contrast-enhanced CT of the chest. Locoregional lymph node (LN) assessment on CI used contrast-enhanced CT or MRI of the relevant anatomic region (neck for head-and-neck primaries, pelvis for genitourinary primaries, abdomen/pelvis for retroperitoneal primaries); ultrasound was used only as an adjunct for superficial nodal basins when clinically indicated and was not used as the principal nodal staging tool.

At KHCC, [^18^F]-FDG PET/CT is utilized as part of initial staging. Although PET/CT provides whole-body metabolic information that is highly sensitive for osteolytic skeletal disease, it does not replace bone marrow aspiration and biopsy, which remain part of standard staging per NCCN and EpSSG recommendations, as PET/CT may miss low-volume or diffusely infiltrative marrow involvement. At our institution, bone scintigraphy is omitted when PET/CT is available; bone marrow aspiration/biopsy is performed at the discretion of the treating team based on clinical and imaging features. Because bone scintigraphy and biopsies were only performed in a small, sporadic set of cases, they were not systematically compared with PET/CT in this study.

For each patient, imaging data were collected for the primary tumor, loco-regional LN, and distant metastases, along with PET/CT SUVmax values. Tumor staging and risk stratification, according to the COG classification, were based on the final multidisciplinary consensus. This decision integrated all available clinical and imaging information rather than relying exclusively on either CI or PET/CT findings. 

Because routine histopathological sampling of all LNs is clinically impractical and challenging in pediatric populations, sensitivity and specificity calculations were considered apparent. For lesions without biopsy confirmation, true disease status was determined using a composite reference standard based on multidisciplinary consensus, integrating baseline imaging findings, clinical follow-up, and treatment response. This approach, while pragmatic, introduces potential verification and incorporation bias, which should be acknowledged when interpreting the reported sensitivity and specificity.

Statistical analyses were performed in R (version 4.4.3). Continuous variables were summarized as medians with interquartile ranges (IQRs), while categorical variables were reported as frequencies and percentages. An SUVmax threshold of ≥3.6 was utilized for survival analysis. This cut-off was determined based on previously published literature demonstrating prognostic relevance in pediatric rhabdomyosarcomas [12]. Survival outcomes were evaluated using the Kaplan–Meier method, and comparisons between survival curves were made using the log-rank test. A *p*-value < 0.05 was considered statistically significant. 

## 3. Results

### 3.1. Patient and Tumor Characteristics 

A total of 56 pediatric patients diagnosed with RMS who underwent [^18^F]-FDG PET/CT as part of their initial evaluation at our center were included in this study. The median age at diagnosis was 5.3 years (range: 0.1–18 years), with approximately two-thirds of the patients (*n* = 36, 64.3%) diagnosed at or before the age of 10 years. Slightly more than half of the cohort were female (*n* = 31, 55%), resulting in a male-to-female ratio of 0.81. 

The most common primary tumor site was head and neck (HN) parameningeal (*n* = 15, 26.8%), followed by genitourinary (GU) sites excluding bladder and prostate (*n* = 10, 17.9%), and HN non–parameningeal non-orbital (*n* = 8, 14.3%). Embryonal histology was the most frequent histologic subtype (*n* = 38, 67.9%). FOXO1 fusion status was assessed in 25 patients (44.6%), with five (8.9%) testing positive and 20 (35.7%) testing negative. Testing was not performed in 31 patients (55.4%); primarily reflecting those diagnosed prior to the routine implementation of molecular testing at our center.

Regarding tumor burden at initial diagnosis, the tumor size exceeded 5 cm in 37 patients (66%), and regional LN involvement was detected in 19 patients (33.9%). Metastatic disease at diagnosis was identified in 11 patients (19.6%). The most common metastatic sites were distant LNs and bone (*n* = 5 each), followed by the lung (*n* = 4). Of the metastatic subgroup, five patients (45.5%) presented with multisite involvement. Complete characteristics and site-specific data are summarized in Table 1.

Clinical risk grouping was determined according to the COG risk stratification system. Patients were classified as low-risk (LR; *n* = 11, 20%), intermediate-risk (IR; *n* = 39, 70%), and high-risk (HR; *n* = 6, 11%). A detailed breakdown of patients and tumor characteristics is presented in Table 1.

Of the cohort, 48 patients (85.7%) had evaluable SUVmax values and were included in the metabolic survival analysis. The remaining eight patients were excluded, either due to a post-operative complete metabolic response (*n* = 7) or missing SUVmax data (*n* = 1). 

### 3.2. Overview of Management and Treatment 

Overall, chemotherapy was administered to all 56 patients. Radiotherapy was delivered to 45 (80%), with a median dose of 50.4 Gy (range: 24–50.4 Gy). Surgical resection of the primary tumor was performed in 25 patients (44.6%) during the course of treatment. Of these 13 underwent upfront surgery and 12 had surgery after neoadjuvant chemotherapy. Negative margins were achieved in 13 of the resected cases (52%).

### 3.3. PET/CT Findings and Concordance with Conventional Imaging 

Out of the 56 patients, 43 underwent neo-adjuvant chemotherapy following diagnostic biopsy without prior surgical intervention. In all 43 of these cases, PET/CT scans successfully identified increased FDG uptake at the primary tumor site. Specifically, PET/CT was positive in the primary site only (*n* = 24), the primary and definite regional LNs (*n* = 10), primary site with suspicious regional LNs (*n* = 2), and the primary site alongside multiple sites (*n* = 7). 

Of the 13 patients (23.2%) who underwent upfront surgery prior to referral, PET/CT showed no abnormal uptake in seven cases (53.8%). Residual uptake at the primary site was observed in three (23.1%), and loco-regional LN involvement was detected in the remaining three cases (23.1%).

Across the entire cohort, locoregional LN involvement was identified by CI in 19 patients (33.9%) and by PET/CT in 19 patients (33.9%), with 17 concordant positives and four discordant cases (two positive on CI only, two positive on PET/CT only). Of the 11 patients with distant metastases detected by CI, PET/CT identified 10 (90.9%). In the single discordant case, a lung metastasis was visible on both the dedicated chest CT and the CT component of the PET/CT, but it did not demonstrate increased FDG uptake on the PET scan. The median SUVmax was 5.4 (range: 1.8–13).

PET/CT detected osseous metastases in 5 patients (8.9%); a concurrent bone scan was obtained in 1 of these 5 patients, and it was concordant. Separately, bone marrow aspiration/biopsy was performed at the discretion of the treating team in 3 patients; 1 biopsy demonstrated marrow infiltration and 2 were negative. PET/CT findings in the corresponding marrow regions were concordant with the biopsy result in all 3 patients. Additionally, PET/CT confirmed a lung metastasis that had initially been deemed suspicious on a chest CT.

Overall, PET/CT findings were largely concordant with CI, with only a few exceptions. In two cases, CI suggested locoregional LN involvement that was not supported by PET/CT; a biopsy was performed in one of these cases and was negative. In another two cases, PET/CT detected nodal disease missed by MRI/CT, which was subsequently confirmed by biopsy in one patient. The final discordant case involved a lung metastasis detected on CI but not visualized on PET/CT; the lesion measured 0.6 cm and was located in the upper lobe of the right lung.

Focusing specifically on LN evaluation and using each modality as a reference for the other, PET/CT identified 17 of the 19 cases considered positive on CI, yielding a sensitivity 89.5%. It also classified 35 of 37 negative cases (specificity 94.6%). Conversely, when using PET/CT as the reference standard, CI identified 17 of 19 cases considered positive (sensitivity 89.5%) and classified 35 of 37 negative cases (specificity 94.6%).

### 3.4. Impact of PET/CT Findings on Management 

In nine patients (16.1%), PET/CT findings directly altered management decisions (Table 2). Radiotherapy was initiated in two patients who would not have otherwise received it. For the first patient, CI showed no LN involvement, but PET/CT identified locoregional LN metastases (subsequently confirmed by biopsy). For the second patient, CI indicated a low suspicion of residual disease following upfront resection; however, PET/CT provided metabolic evidence of residual disease (also confirmed by biopsy), prompting the initiation of radiotherapy. In some cases (5/9), the biopsy results were the final basis for decisions regarding RT, not PET alone.

Additionally, PET/CT findings led to radiation field modifications in seven patients:

- Field Reductions (*n* = 3): The radiation field was reduced in three patients due to PET/CT downstaging. Two patients had suspicious LNs on CI that were negative on PET/CT (one confirmed by biopsy). A third patient had positive LNs on CI that showed low suspicion on PET/CT (also confirmed negative by biopsy).
- Field Widenings (*n* = 4): in all four patients, the additional volume treated corresponded to the PET-positive nodal basin delineated on the co-registered low-dose CT in consensus with the nuclear medicine physician and radiation oncologist. Patient 1 had disease confined to the primary site on CI; PET/CT identified an ipsilateral regional lymph node. Biopsy performed and was positive. The field was extended to include the PET-positive nodal basin. Patients 2–4 had lymph nodes deemed low-suspicion on CI but highly suspicious or metastatic on PET/CT. Biopsies were not done; management proceeded on integrated imaging plus multidisciplinary review. In all three, the irradiated volume was the PET-positive nodal basin rather than an enlargement to a CI-suspicious but PET-negative node.

PET/CT altered the planned management—most often the radiation therapy target volume—in 9/56 patients (16.1%, 95% CI 7.6–28.3%). The yield was highest in IRS clinical group III (8/34, 23.5%; 95% CI 10.7–41.2%) and in non-metastatic patients (9/45, 20%; 95% CI 9.6–34.6%), Table 3.

### 3.5. Follow-Up and Outcomes 

Over a median follow-up period of 33.3 months (range: 2–109 months), 19 patients (33.9%) experienced a disease-related event. Eighteen patients (32.1%) died (median time to death: 16.5 months). Two patients experienced disease progression, and 17 experienced recurrence. Recurrences were primarily local (*n* = 8), followed by LNs, lung, and central nervous system involvement (*n* = 3 each). 

**Figure 1:**
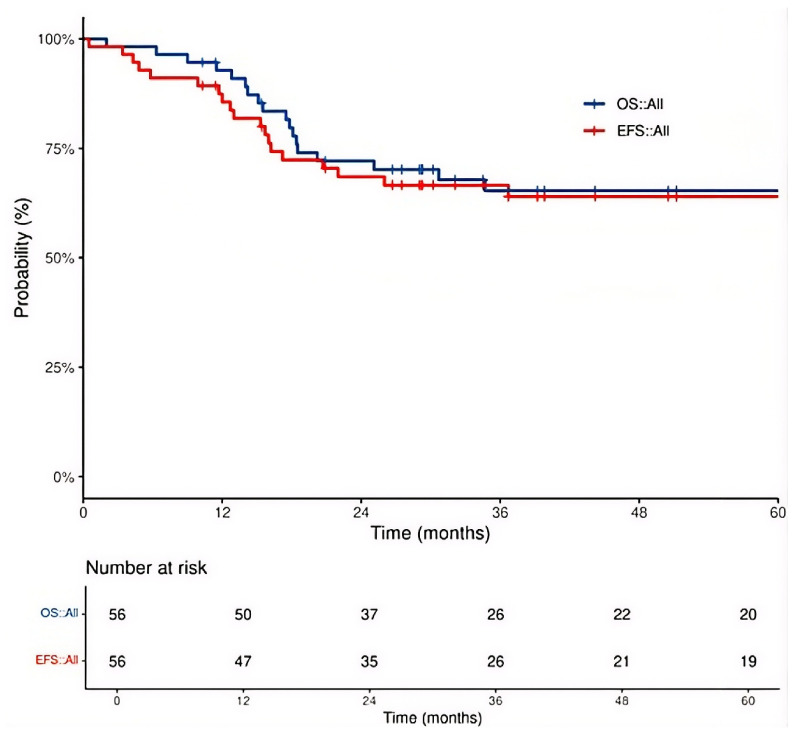
Kaplan–Meier Survival Analysis for the Total Patient Cohort.

**Figure 2:**
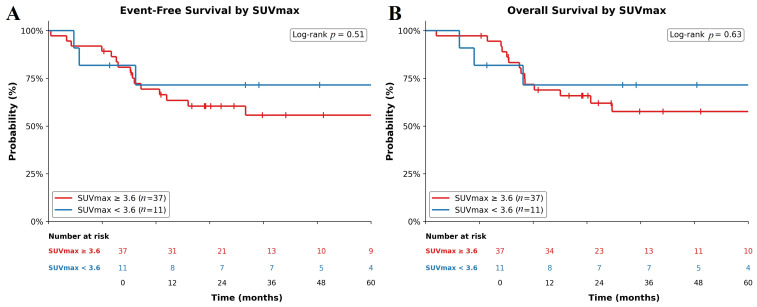
Kaplan–Meier analysis of survival based on a SUVmax cut-off of 3.6. (**A**). Event-Free Survival: Probability of event-free survival for the Low SUVmax group (<3.6, blue) vs. the High SUVmax group (≥3.6, red). (**B**). Overall Survival: Probability of survival for the Low SUVmax group (<3.6, blue) vs. the High SUVmax group (≥3.6, red).

The cohort’s 3-year event-free survival (EFS) and overall survival (OS) rate were 65.3% and 66.5%, respectively (Figure 1). In a pre-specified exploratory analysis using a previously published threshold, patients with a baseline SUVmax ≥3.6 had numerically lower 3-year EFS (57.6% vs. 71.6%; *p* = 0.51; Figure 2A) and OS (60.4% vs. 71.6%; *p* = 0.63; Figure 2B), though neither reached statistical significance. 

## 4. Discussion

Accurate diagnosis and staging are critical for guiding management and optimizing outcomes in pediatric RMS. [^18^F]-FDG PET/CT has emerged as a valuable adjunct to CI, providing metabolic information that enhances assessment of disease burden and informs therapeutic planning [8,9,13]. Its utility is further supported by studies in other sarcomas, where FDG uptake correlates with tumor proliferation and aggressiveness, highlighting its potential prognostic value [14,15,16].

Building on its recognized utility in assessing disease burden, PET/CT has also been shown to substantially improve overall staging in pediatric RMS. Historically, Tateishi et al. reported an overall staging accuracy of 86% for PET/CT compared with 54% for CI, with upstaging observed in approximately 23% of patients [17]. However, this comparison must be interpreted with caution, as the quality of both CI (particularly MRI protocols) and PET/CT has improved substantially over the intervening years; staging accuracy differences reported in older studies may not be reproducible with contemporary imaging technology. In our cohort, however, PET/CT did not substantially change formal overall staging and no patients were upstaged. Contemporary studies suggested that upstaging primarily occurs when PET/CT detects skeletal lesions missed on bone scintigraphy [8,12,18]. At our institution, to minimize radiation exposure and the burden of multiple staging examinations on pediatric patients, baseline bone scintigraphy is systematically omitted when a whole-body [^18^F]-FDG PET/CT is obtained. While the absence of concurrent bone scans limited our ability to document formal skeletal upstaging via PET/CT in this specific cohort, this streamlined approach reflects an evolving, patient-centric clinical paradigm. It demonstrates that PET/CT efficiently consolidates systemic staging without compromising diagnostic yield. 

Although overall staging changes were limited, PET/CT demonstrated particular strength in nodal assessment. In our study, PET/CT altered nodal staging in seven patients, demonstrating an apparent sensitivity of 89.5% and a specificity of 94.6% relative to CI. Because not all nodes were biopsied, these values are considered apparent; however, histologic confirmation in sampled nodes verified that PET-negative sites were true negatives, and suspicious findings on CI not confirmed by PET/CT were ultimately benign. These results are consistent with prior reports in which PET/CT consistently outperformed CI for locoregional and distant nodal evaluation. For example, Mercolini et al. reported PET/CT sensitivities of 96.2% for locoregional nodes and 94.8% for distant nodes, Federico et al. observed 94% sensitivity for PET/CT versus 49% for CI, and Eugène et al. demonstrated 100% sensitivity for PET/CT versus 75% for CI [8,9,19]. Collectively, these findings underscore PET/CT as a robust tool for nodal evaluation, while reaffirming that histopathologic confirmation remains essential for definitive staging when feasible. We emphasize that the apparent sensitivity and specificity values reported should be interpreted with caution, as they are subject to verification bias (only clinically selected nodes were biopsied) and incorporation bias (PET/CT finding contributed to the composite reference standard). The true diagnostic accuracy of PET/CT for nodal staging in pediatric RMS can only be determined through prospective studies with systematic histopathologic confirmation.

In the four discordant nodal cases in our cohort, the clinical decision ultimately rested on histopathologic confirmation rather than on PET/CT findings alone. This reinforces that PET/CT, despite its high apparent sensitivity and specificity, does not eliminate the need for tissue diagnosis in equivocal cases and may generate additional procedures with their attendant risks, including anesthesia and incremental radiation exposure from image-guided biopsies.

While our study reports a high apparent sensitivity (89.5%) and specificity (94.6%) for nodal detection, we acknowledge the inherent verification bias common in pediatric cohorts where systematic lymphadenectomy is not standard of care. However, our findings align with the superior sensitivity reported by Federico et al. (94%) and Mercolini et al. (96.2%) [8,9], reinforcing that PET/CT often detects metabolically active disease in nodes that do not yet meet the size criteria for malignancy on conventional CT or MRI. This early detection is pivotal for accurate risk stratification in RMS. 

Beyond nodal assessment, PET/CT effectively identified skeletal metastases, detecting involvement in five patients. Although baseline bone scintigraphy was not performed in our cohort, the broader literature consistently demonstrates PET/CT’s superior sensitivity for osteolytic lesions typical of RMS. For instance, PET/CT sensitivities have been reported as 86% to 100% compared to 63% to 67.9% for bone scan. Similarly, Ricard et al. identified 11 lesions in PET/CT compared with 3 on bone scans, and Federico et al. detected all osseous metastases versus partial detection on bone scans [8,9,17,18,19]. These comparisons highlight PET/CT’s reliability in detecting skeletal involvement.

In contrast, PET/CT performance was more limited in pulmonary staging, particularly for small nodules. In our cohort, a 0.6 cm nodule detected on chest CT in one patient was not visualized on PET/CT. This limitation can be attributed to several well-recognized technical and biological factors, including partial volume effects in sub-centimeter lesions, respiratory motion causing blurring and underestimation of tracer uptake, and the inherently low glycolytic activity of some small pulmonary metastases [20]. This limitation is well-documented: Mercolini et al. reported CT sensitivity of 100% versus 79.6% for PET/CT, Federico et al. noted that PET/CT missed three of seven nodules; and El-Kholy et al. highlighted reduced detection for sub-centimeter lesions [8,9,12,13]. Inherent limitations in spatial resolution, partial-volume effects, and low FDG metabolic activity of sub-centimeter lesions likely account for these false negatives, emphasizing the continued necessity of thin-slice chest CT for accurate pulmonary evaluation [8,21,22].

It is important to contextualize these findings within the EpSSG RMS2005 framework, which classifies small pulmonary nodules (≤ four nodules <5 mm, or one nodule 5–10 mm) as “indeterminate” [23]. Patients with indeterminate nodules in the absence of other metastases are treated as having localized disease and histopathological confirmation is not recommended as it does not change management or survival. Thus, the clinical impact of PET/CT’s inability to detect such sub-centimeter nodules may be limited within current treatment paradigms.

Beyond staging, PET/CT influenced treatment planning in a substantial proportion of our cohort. Clinical management was modified in 16.1% of cases (*n* = 9), primarily through adjustments to radiotherapy. Specifically, PET/CT findings led to radiation field expansions in four patients, field reductions in three patients, and the initiation of definitive radiotherapy in two patients. Our observations align closely with prior studies, which report that PET/CT influenced management in 13% to 17% of patients, most commonly via radiotherapy planning adjustments [19,21,24]. Ultimately, these findings illustrate that PET/CT exerts a direct and clinically meaningful impact on therapeutic decision-making, even in contemporary cohorts undergoing comprehensive CI evaluation. Formal quantification with exact confidence intervals confirmed that PET/CT altered planned management in approximately one in six evaluable patients, with the highest yield in clinical group III disease. While the wide confidence intervals reflect the modest sample size, these estimates support the clinical impression that PET/CT’s diagnostic utility in pediatric RMS is concentrated in patients whose locoregional disease burden is most amenable to radiation field modification. 

PET/CT also provided insights into potential prognostic markers. In our study, SUVmax values ≥3.6 were associated with trends toward poorer OS and EFS, although statistical significance was not reached. While SUVmax is widely used and clinically practical, it reflects only focal tumor metabolism and may not fully capture total disease burden. Advanced volumetric PET parameters, such as metabolic tumor volume (MTV) and total lesion glycolysis (TLG), are emerging as complementary biomarkers; however, their role in pediatric rhabdomyosarcoma remains to be fully established and was beyond the scope of the present study [25,26]. Our study, with a modest cohort (*n* = 56) and the shortest follow-up duration (median 33.3 months), may have been underpowered to detect survival associations, similar to the findings of El-Kholy et al. (*n* = 98, median follow-up 36 months) [12]. By contrast, larger studies with longer follow-up periods, such as Casey et al. (*n* = 107, median follow-up 64 months), have successfully demonstrated statistically significant associations between SUVmax and survival outcomes [11]. Differences across studies likely reflect variations in sample size, follow-up duration, RMS subtype distribution, and the specific SUVmax thresholds utilized. Future multicenter studies incorporating volumetric PET parameters, such as metabolic tumor volume and total lesion glycolysis, are needed to clarify prognostic utility of PET/CT in this setting.

A central, still-unresolved question is whether [^18^F]-FDG PET/CT, for all its staging utility, is a validated prognostic or predictive tool in pediatric RMS. To date, no prospective or randomized trial has demonstrated that staging or risk stratification based on PET/CT, including SUVmax or other metabolic parameters, improves event-free or overall survival. Available evidence, including our own, shows that PET/CT refines anatomic staging and alters therapeutic planning in a meaningful minority of patients, but whether these refinements actually lead to better outcomes remains unknown. Our SUVmax analyses should therefore be interpreted as exploratory and hypothesis-generating, consistent with current EANM pediatric guidance and contemporary reviews, which explicitly caution against treating SUVmax as a stand-alone, independent prognostic marker. Prospective, cooperative-group studies with harmonized acquisition protocols, pre-specified thresholds, and volumetric endpoints (MTV, TLG) are required before PET-derived metrics can be incorporated into risk-adapted therapy.

An important consideration that must accompany any discussion of PET/CT utility in pediatric oncology is the additional radiation burden it imposes. A typical whole-body [^18^F]-FDG PET/CT examination delivers an effective dose of approximately 5–15 mSv to a pediatric patient, depending on weight, scanner, and low-dose CT protocol. This is additive to the radiation from contrast-enhanced diagnostic CT scans already required for staging. In a population with decades of expected survival, the cumulative risk of radiation-induced secondary malignancy is a non-trivial concern. Furthermore, PET/CT is not routinely recommended for long-term surveillance in pediatric RMS: most relapses are detected clinically or by MRI/CT, and no evidence demonstrates that PET/CT-based surveillance improves EFS or OS [21]. The decision to incorporate PET/CT at diagnosis should therefore be weighed against these radiation costs, and its use should be limited to scenarios where it is expected to change management, as it did in 16.1% of our cohort. 

This study has several limitations. The retrospective, single-center design and modest sample size may limit generalizability of the findings. Furthermore, the omission of baseline bone scintigraphy at our institution—consequent to the adoption of PET/CT—precluded a direct head-to-head comparison between PET/CT and traditional technetium-based imaging for skeletal staging. The absence of volumetric PET parameters restricts a more comprehensive assessment of tumor burden and its true prognostic potential. Additionally, the sensitivity and specificity for nodal involvement must be considered apparent, as histologic confirmation was only obtained for a subset of clinically selected patients. The cohort was dominated by embryonal histology (67.9%), with only 11 patients (19.6%) presenting with the more aggressive alveolar subtype. Given that alveolar RMS often exhibits higher baseline SUVmax and carries a poorer prognosis, our survival analysis may have been underpowered to reach statistical significance (*p* = 0.5 for OS). Additionally, we did not quantify the cumulative radiation exposure from PET/CT in our cohort or evaluate the incremental dose relative to standard CI. Given that the majority of our patients are long-term survivors, the additional radiation burden from PET/CT is a clinically relevant consideration that warrants formal dosimetric evaluation in future studies. Formal RECIST response assessment was not feasible in this retrospective cohort because baseline cross-sectional imaging modality varied across patients and pediatric RMS response is conventionally defined by IRS clinical group reassessment rather than by RECIST. Furthermore, the small cohort and low number of events (*n* = 19) precluded a robust multivariable Cox regression. Incorporating covariates such as the Oberlin score, histologic subtype, or treatment modalities would be statistically underpowered and highly prone to over-fitting. Consequently, the independent prognostic value of SUVmax remains exploratory and requires validation in adequately powered, multicenter studies. 

## 5. Conclusions

[^18^F]-FDG PET/CT is a valuable diagnostic adjunct in pediatric RMS, providing an accurate assessment of nodal and skeletal disease and influencing therapeutic planning. While its superior sensitivity in these areas is clear, its limitations in pulmonary staging highlight the ongoing necessity of thin-slice chest CT. SUVmax has not been prospectively validated as an independent prognostic or predictive biomarker in pediatric RMS; the trends observed here are exploratory and should not inform clinical decision-making. Prospective, multicenter studies incorporating volumetric PET metrics are required before any prognostic role can be established. Overall, our findings support the routine integration of PET/CT into clinical pathways for pediatric RMS and emphasize the importance of multicenter validation in the region to optimize staging and management protocols.

## Figures and Tables

**Table 1:** Characteristics of Patients and Tumor in all Groups.

Variable	All Patients, Number (Percentage)
Total Patients	56
Age at Diagnosis Median (range) years	5.3 (0.1–18)
Sex	
Females	31 (55%)
Males	25 (45%)
Primary Tumor Category	
Head and neck Parameningeal	15 (26.8%)
Head and neck (Non-orbital Non-Parameningeal)	8 (14.3%)
Orbital	5 (8.9%)
Genitourinary (excluding bladder/prostate)	10 (17.9%)
Extremity	7 (12.5%)
Others	11 (19.6%)
Histologic Subtypes	
Embryonal	38 (67.9%)
Alveolar	11 (19.6%)
NOS	7 (12.5%)
TNM Stage	
Stage 1	24 (43%)
Stage 2	7 (13%)
Stage 3	14 (25%)
Stage 4	11 (20%)
IRS Clinical Group	
I	2 (3.6%)
II	9 (16%)
III	34 (61%)
IV	11 (20%)
FISH FOXO1 status	
Positive	5 (8.9%)
Negative	20 (35.7%)
Not performed	31 (55.4%)
Risk group	
Low Risk	11 (20%)
Intermediate Risk	39 (70%)
High Risk	6 (11%)
Tumor Size	
≤5 cm	19 (34%)
>5 cm	37 (66%)
Metastasis	
Yes *	11 (19.6%)
Distant LNs	5
Bone	5
Lung	4
Others (bone marrow, peritoneal, breast)	3
No	45 (80.4%)
Treatment	
Surgery	25 (44.6%)
Upfront	13 (52%)
Delayed	12 (48%)
Radiation therapy	45 (80%)

Abbreviations, NOS, none otherwise specified; TNM, tumor node metastasis; IRS, international rhabdomyosarcoma study; LN, lymph nodes; FISH, Fluorescence In Situ Hybridization. * Some patients have more than one metastatic site.

**Table 2:** Impact of PET/CT Findings on Radiotherapy Management Modifications and Pathological Correlation.

Management Modification	Patient	Conventional Imaging Findings	PET/CT Findings	Pathological Confirmation
Radiotherapy Initiated (*n* = 2)	P1	No LNs involvement	Positive LNs	Positive
	P2	Low suspicion of residual disease post-surgery	Metabolic evidence of residual disease	Positive
Radiation Field Reduced (*n* = 3)	P3	Suspicious LNs	Negative LNs	Not performed
	P4	Suspicious LNs	Negative LNs	Negative
	P5	Positive LNs	Low suspicion LNs	Negative
Radiation Field Widened (*n* = 4)	P6	No LNs involvement	Positive LNs involvement	Positive
	P7	Low suspicion LNs	Highly suspicious/metastatic LNs	Not performed
	P8	Low suspicion LNs	Highly suspicious/metastatic LNs	Not performed
	P9	Low suspicion LNs	Highly suspicious/metastatic LNs	Not performed

Abbreviations: *n*, number; PET/CT, Positron Emission Tomography/Computed Tomography; LN, lymph node.

**Table 3:** Proportion of Patients in Whom PET-CT Changed Planned Management, with Exact 95% Confidence Intervals. Management Change Consisted Predominantly of Radiation Therapy Field Modification.

Stratum	Subgroup	N Evaluable	n Changed	Proportion (95% CI)
Overall cohort	All evaluable	56	9	16.1% (7.6–28.3%)
By risk group	Low Risk	11	1	9.1% (0.2–41.3%)
	Intermediate Risk	39	8	20.5% (9.3–36.5%)
	High Risk	6	0	0.0% (0.0–45.9%)
By IRS clinical group	I	2	0	0.0% (0.0–84.2%)
	II	9	1	11.1% (0.3–48.2%)
	III	34	8	23.5% (10.7–41.2%)
	IV	11	0	0.0% (0.0–28.5%)
By metastatic status	Non-metastatic	45	9	20.0% (9.6–34.6%)
	Metastatic	11	0	0.0% (0.0–28.5%)

## Data Availability

The data presented in this study are available on request from the corresponding author due to ethical restriction.
